# Octopus sucker-inspired, Turkish gall extract-integrated microneedle patch for oral ulcer mitigation

**DOI:** 10.1016/j.isci.2026.115557

**Published:** 2026-04-01

**Authors:** Lin Lin, Shilin Guo, Cheng Zhao, Xiaotan Dou, Wei Han, Chuanhui Song

**Affiliations:** 1 Nanjing Stomatological Hospital, Affiliated Hospital of Medical School, Institute of Stomatology, Nanjing University, Nanjing 210008, China; 2 Department of Gastroenterology, Nanjing Drum Tower Hospital, The Affiliated Hospital of Medical School, Nanjing University, Nanjing 210008, China

**Keywords:** Pharmacology, Biomedical engineering, Materials science

## Abstract

Despite pharmacological advancements of oral ulcer treatment, developing sustained-release delivery systems remains a challenge. To address this, we designed a bioinspired microneedle platform integrating Turkish gall extract for targeted ulcer therapy. Drawing on the structural principles of octopus suckers, we fabricated a 3D-printed microneedle array with suction-enhanced morphology, replicated into a hydrogel-based mold. The microneedle matrix was synthesized by combining Turkish gall extract, polydopamine, and hyaluronic acid methacrylate, leveraging polydopamine’s catechol chemistry for robust mucoadhesion. Pharmacological evaluations confirmed the dual functionality of the herbal component, demonstrating dose-dependent anti-inflammatory and anti-oxidant effects *in vitro* and in rodents. Applied to oral ulcer lesions in rats, the microneedle system exhibited prolonged mucosal retention with sustained drug release, significantly accelerating epithelial regeneration. These findings establish the sucker-inspired herbal microneedle platform as a mechanochemically optimized therapeutic strategy and provide a translational framework for modernizing traditional herbal delivery.

## Highlights

• Octopus sucker-inspired microneedle patch with enhanced oral mucosal adhesion
• Turkish gall extract confers anti-inflammatory and anti-oxidant dual functionality
• Sustained drug release at ulcer site accelerates epithelial regeneration in rats
• Bioinspired platform integrates herbal medicine with mechanochemical design

## Graphical abstract

**Figure:**
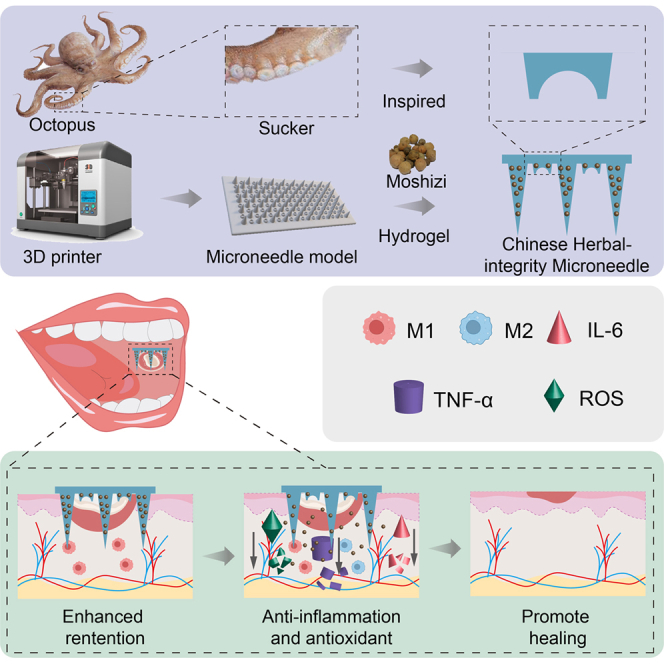

## Introduction

Oral ulcers represent a prevalent oral mucosal disorder characterized by localized lesions, often accompanied by significant pain that impairs mastication and social functioning.^1^^,^^2^^,^^3^ Current therapeutic strategies primarily employ topical formulations, such as pharmaceutical films and fast-dissolving tablets, for glucocorticoid delivery to accelerate wound healing.^4^^,^^5^ While glucocorticoids (e.g., dexamethasone) effectively suppress local inflammatory responses and promote tissue regeneration, their clinical utility is limited by gastrointestinal complications and increased risks of infection associated with immunosuppression.^6^^,^^7^^,^^8^ Despite extensive research efforts to identify hormone alternatives, satisfactory substitutes remain elusive.^9^ Furthermore, the moist and dynamic oral environment substantially compromises the efficacy of conventional dosage forms, resulting in transient drug retention and subtherapeutic concentrations.^10^^,^^11^ These limitations necessitate frequent dosing regimens that inevitably contribute to increased medication waste and patient non-compliance, highlighting the urgent need for sustained-release delivery systems incorporating alternative anti-inflammatory agents.

**Figure 1:**
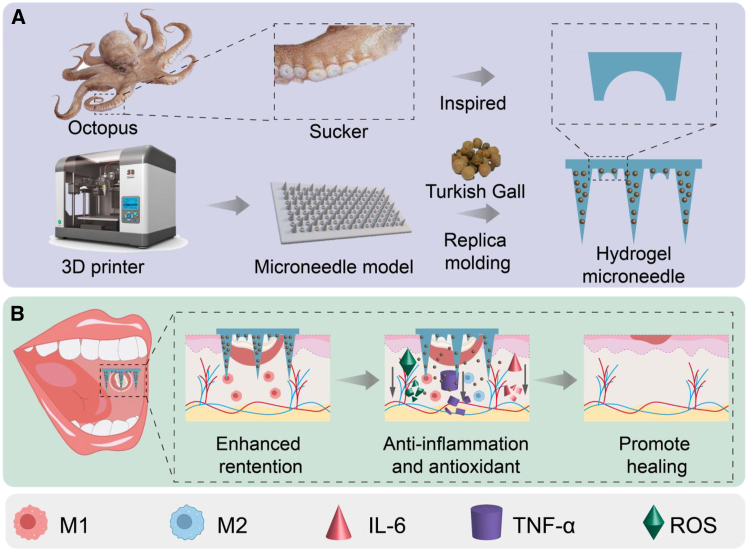
The design of an octopus sucker-inspired Chinese herbal-integrated microneedle patch (A) Synthesis of the Chinese herbal-integrated microneedle patch. (B) Application of the Chinese herbal-integrated microneedle patch.

To address these challenges, we developed a bioinspired Chinese herbal-integrated microneedle patch for enhanced oral ulcer therapy (Figure 1). Turkish gall, a traditional Chinese medicinal herb, was selected for its documented anti-inflammatory and anti-oxidant properties.^12^^,^^13^ Notably, Turkish gall-based formulations, such as the Xipayi gingival rinse, have been clinically validated for oral ulcer management.^14^^,^^15^^,^^16^ The Turkish gall extract (TGE) primarily contains phenolic compounds, which exhibit wound-healing, astringent, anti-bacterial, anti-inflammatory, and anti-ulcer properties.^12^ The microneedle platform synergistically combines minimally invasive transdermal delivery with structural optimization to achieve enhanced tissue adhesion and drug-loading capacity.^17^^,^^18^^,^^19^^,^^20^^,^^21^^,^^22^^,^^23^^,^^24^^,^^25^ Also, related research has proved that the microneedles applied to the mucosal drug delivery are highly effective, minimally invasive, locally enhanced, and locally retained.^26^^,^^27^ Drawing inspiration from octopus sucker morphology,^28^^,^^29^ we engineered a suction-enhanced microneedle array capable of prolonged mucosal retention through combined physical and chemical adhesion mechanisms. This innovative design integrates TGE with a polydopamine-hyaluronic acid methacrylate (HAMA) composite matrix, endowing the system with exceptional wet-adhesion properties critical for oral cavity applications.

In this study, we constructed a 3D print-based sucker hydrogel microneedle patch to deliver TGE into the ulcer wound, thereby reducing inflammation and accelerating tissue healing. Inspired by the octopus sucker, we designed and fabricated a suction cup structure on the microneedle patch, which enhances the retention capacity of the microneedle patch through physical action. With mixing of the TGE, polydopamine, and HAMA, the microneedle patch acquires excellent wet-adhesion properties via the chemical structure of the polydopamine.^30^ The resultant microneedle patch has improved the adhesive ability, making the microneedle patch adhesive in moist and wet environments. Comprehensive *in vitro* and *in vivo* evaluations confirmed the dual functionality of the integrated TGE, demonstrating potent anti-inflammatory and anti-oxidant activities. When applied to rat oral ulcer models, the patch maintained sustained drug release at the lesion site, effectively promoting tissue regeneration and epithelial repair. These findings establish the octopus sucker-inspired herbal microneedle system as a promising therapeutic alternative for managing oral ulcers, while providing insights into the modernization of traditional herbal medicine delivery platforms.

## Results

### Fabrication and characterization of the microneedle patch

**Figure 2:**
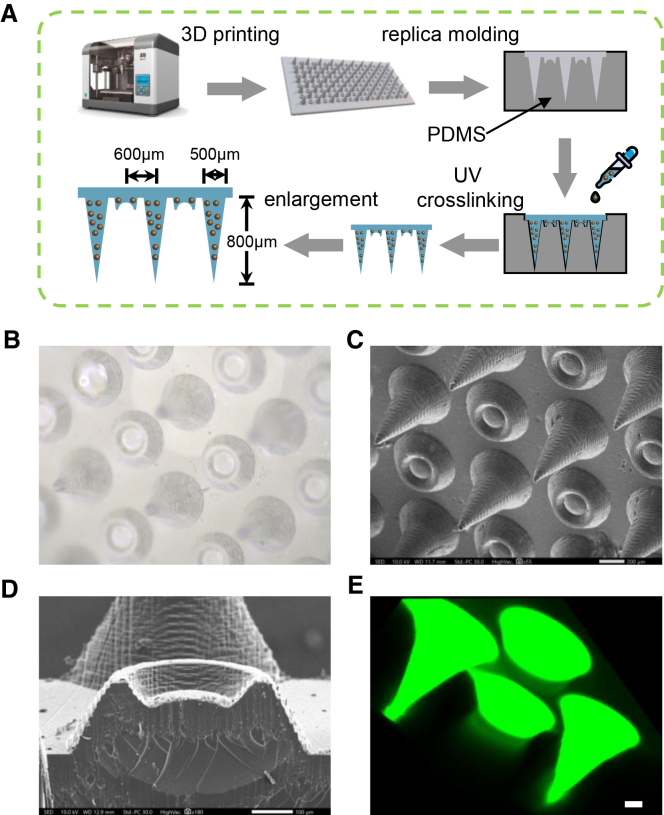
Characterization of the sucker-integrated microneedle patch (A) The process of preparation of the microneedles. (B) Digital picture of the hydrogel microneedle patch. (C) Image from SEM. (D) Cross-section of the sucker on the patch. (E) 3D reconstruction of the microneedle patch with fluorescent dye. Scale bars, 100 μm.

Inspired by the natural adhesion of the octopus sucker, we integrated the sucker structure into microneedle patches to enhance retention in oral lesions. We used a 3D printer to synthesize microneedles composed of light-cured resin, obtaining the expected microneedles (Figure 2A). Then, we created a native model to develop hydrogel microneedle patches from the polydimethylsiloxane (PDMS). To determine the optimal HAMA concentration, we evaluated the mechanical properties of the hydrogels and found that a 5% formulation exhibited a more suitable Young’s modulus for microneedle fabrication (Figure S1A). Moreover, a 7.5% solution presented handling difficulties during mold filling due to its high viscosity. In contrast, the incorporation of TGE did not significantly alter the mechanical performance of the hydrogel system (Figure S1B). The resultant hydrogel microneedle patch is shown in Figure 2B; the tips and suckers were alternatively distributed one by one. Electron scanning microscopy was used to observe the microneedle patch’s microstructure directly. The hydrogel resembles the 3D printed microneedle, as regular and orderly print marks can be seen (Figure 2C). The structure and length of the needle tip and the suction cup were found to be uniform, respectively. The cross-section of the suction cup under the electron microscope was well demonstrated, showing that the suction cup we obtained fits very well with our original design (Figure 2D). Also, the fluorescent dye was used to show the structure of the microneedle patches (Figure 2E). The cross-section proved the integrity of the tips and the suckers in the patches (Figures S2A and S2B). These data indicated that the microneedle patch containing suckers was successfully produced.

### Enhanced tissue retention and drug delivery performance of the sucker patch

**Figure 3:**
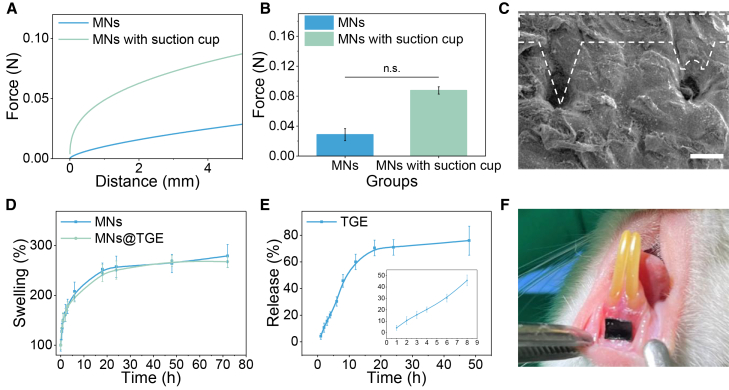
The properties of the microneedle patch involve adhesion and drug release (A) The adhesive force profile of the microneedle with or without the sucker. (B) The maximum force of the microneedle. (C) SEM image of the pig skin after the microneedle application. Scale bars, 200 μm. (D) The swelling profile of the microneedle with/without Chinese herbal. (E) The release profile of the inner TGE from the microneedle patch. (F) The digital picture of the microneedle applied in the rat oral cavity. Data are represented as the mean ± SD. *n* = 3, n.s., not significant.

As the suckers can make the octopus’s adhesion sturdy underwater, we wonder if the suckers patch can have enhanced tissue retention. We used conventional microneedles as the control group to test the power of peeling-off pork skin. As shown in Figure 3A, the sucker patches have enhanced adsorption capacity to cope with pulling. Compared with the conventional microneedles, the adhesive force of sucker patches was much higher (Figure 3B). To confirm the penetration of the microneedles, the pork shin was used as the model tissue. After removing the microneedles, the signs of the tips and the sucker cup were left on the skin, indicating the penetration of the microneedles and the shape of the suction cup (Figure 3C). The strength is relatively small than that reported in other studies,^27^ but it still meets the requirements. We also tested the actual penetration depth, using *ex vivo* rat oral mucosa (Figure S2D). We further tested the swelling ratio of the hydrogel to observe the influence of the TGE on the microneedles. The results showed that there was no noticeable difference in the swelling property between the hydrogel with and without TGE (Figure 3D). The TGE inside the microneedles could release gradually (Figure 3E). Additionally, artificial saliva could accelerate the release due to enzymatic activity. The low pH could also enhance the TGE release (Figure S2E). To confirm drug distribution of the microneedle, fluorescence images and quantitative analysis of fluorescein sodium distribution were obtained in rat oral mucosal tissues following different treatments. In tissues with intact epithelium, superficial fluorescence was observed after application of fluorescein sodium powder (Figure S3A), while the microneedle group (Figure S3B) exhibited enhanced penetration into the lamina propria. In tissues with disrupted epithelium (simulating ulcer conditions), both groups showed deeper fluorescence distribution, but the microneedle-treated group (Figure S3D) demonstrated stronger and more extensive fluorescence signals within the lamina propria than the powder-treated group (Figure S3C). A cross-sectional view (Figure S3E) visually confirmed the deeper horizontal distribution of fluorescence following microneedle application. Quantitative analysis revealed that the microneedle group yielded significantly higher fluorescence intensity in the lamina propria than the powder group, both in tissues with intact (Figure S3F) and disrupted epithelium (Figure S3G) (∗*p* < 0.001). These results indicated that microneedles facilitate more effective deep-tissue delivery of the model drug into the mucosal layer than topical powder application. The final application scenario is shown in Figure 3F.

### *In vitro* biocompatibility, anti-oxidant, and pro-angiogenic effects

**Figure 4:**
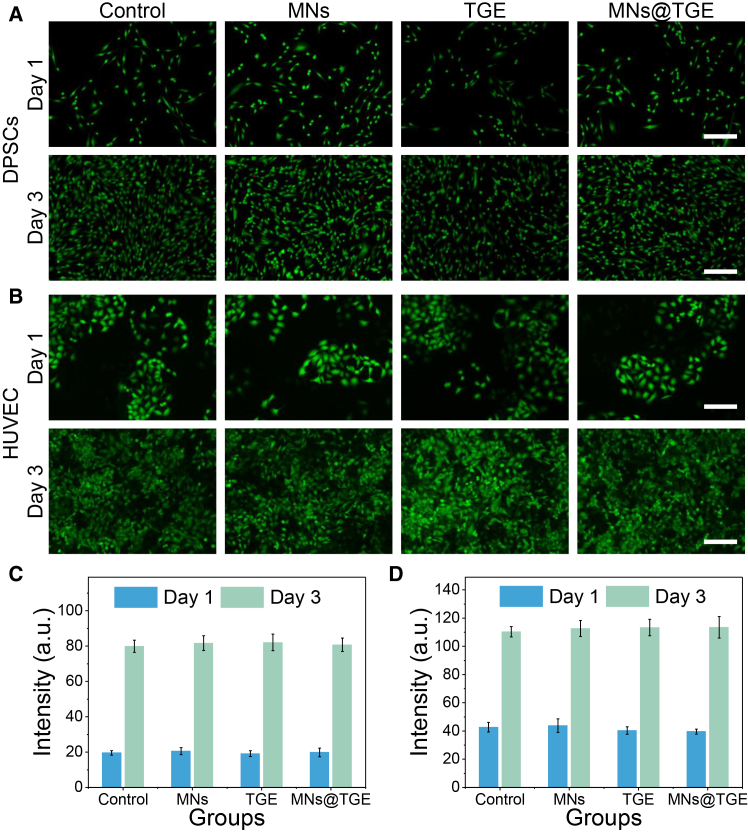
*In vitro* biosafety of the microneedle patch (A) Fluorescence staining of the DPSCs co-cultured with the microneedle for 3 days. (B) Fluorescence staining of the HUVECs co-cultured with the microneedle for 3 days. (C) The quantitated data of (A). (D) The quantitated data of (B). DPSCs, dental pulp stem cells; HUVECs, Scale bars, 100 μm. Data are represented as the mean ± SD. *n* = 3; ∗*p* < 0.05; ∗∗*p* < 0.01.

**Figure 5:**
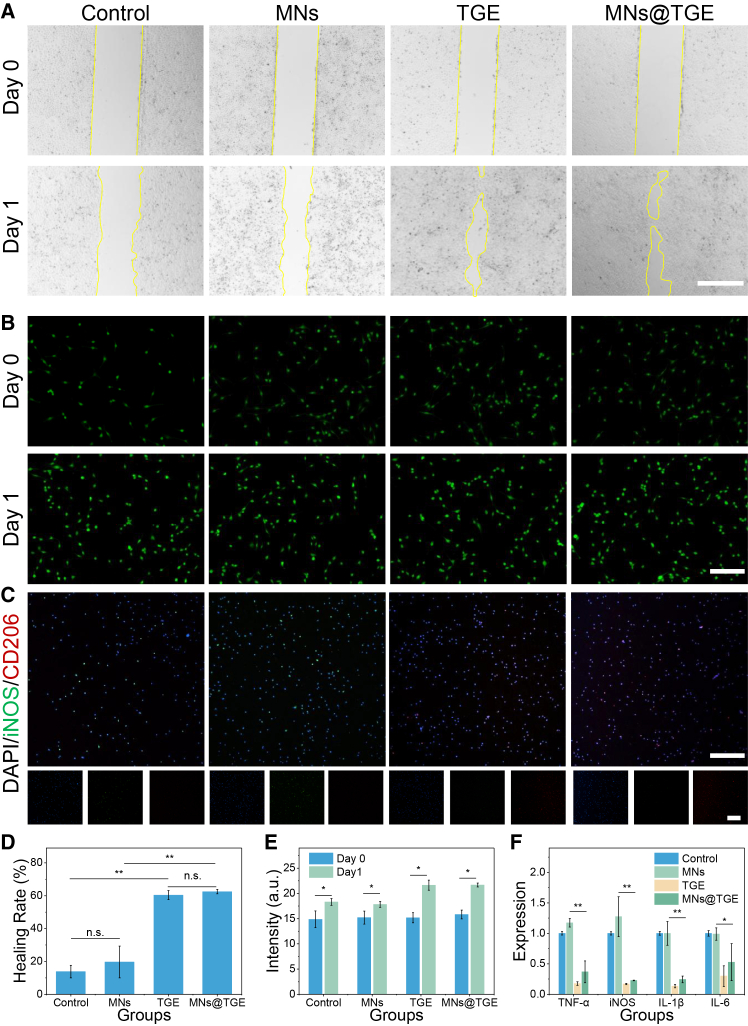
The cellular anti-inflammatory property of the microneedle (A) Scratch test of the HUVECs with different treatments. (B) Biocompatibility of the microneedle with the RAW264.7 cells. (C) CD206 and iNOS immunofluorescent staining of the Raw264.7 cells in different groups. (D) The quantitative analysis of (A). (E) The quantitative analysis of (B). (F) Relative gene expression of the cytokines of the Raw264.7 cells. Data are represented as the mean ± SD. Scale bars, 100 μm. *n* = 3; ∗*p* < 0.05’ ∗∗*p* < 0.01.

The biocompatibility of a biomaterial is the key to its application when used *in vivo*. UV-vis spectroscopy analysis confirmed that the prepared TGEs are consistent with the literature reports (Figure S2C).^13^^,^^31^ A representative HPLC chromatogram of the TGE is provided in Figure S2F. The analysis confirmed the presence of major characteristic peaks consistent with known active constituents (gallic acid). First, we used the *in vitro* cell experiments to check biosafety of the resultant microneedles. The dental pulp stem cells (DPSCs) and endothelial cells were cultured for three days with the microneedles and other groups. The live cell staining showed that the morphology of the cells was sound, and the cell proliferation was in good condition (Figures 4A and 4B). The quantified data were consistent with the fluorescent images (Figures 4C and 4D), and the cells that grew on the hydrogel were observed by the scanning electron microscopy (SEM), indicating the adhesion of the cells (Figure S4A). The CCK-8 and MTT assays were used to evaluate the cytotoxicity of the resultant microneedles, showing the wonderful biosafety (Figures S4B–S4E). The ulcer site had high reactive oxygen species (ROS) content, which could inhibit wound healing. The TGE could decrease the cellular ROS level in a concentration-dependent manner (Figure S5A). The exorbitant ROS could also inhibit cell migration, prolonging the healing process.^32^ The TGE accelerated the cellular migration (Figure S5B), and the TGE-integrated microneedle patch promoted the migration of endothelial cells (Figures 5A, 5D, and S4C). The tube-forming test proved that the tube-forming ability of endothelial cells was enhanced with the TGE (Figure S6).

### *In vitro* anti-inflammatory effects of the TGE-integrated microneedle patch

The inflammation reaction plays a key role in healing the ulcer wound, and it should be regulated to enhance the process. The co-culture results showed that the TGE and the microneedle patch have minimal effect on RAW 264.7 cells (Figures 5B and 5E). To confirm the anti-inflammatory effect of the TGE-integrated microneedle patch, the type I macrophage derived from Raw264.7 cells was divided into different groups. After being cultured for two days, iNOS and CD206^33^ were stained to evaluate the inflammation of the macrophage (Figure 5C). The group of the microneedle with TGE had the highest CD206 expression, indicating the strongest anti-inflammatory effect (Figure S7). To further evaluate the inflammation reaction at the cellular level, the proinflammatory cytokines were tested using qPCR. The inflammation-related cytokines were found to be decreased in the TGE and MNs@TGE groups (Figure 5F).

### *In vivo* wound-healing efficacy in an oral ulcer model

**Figure 6:**
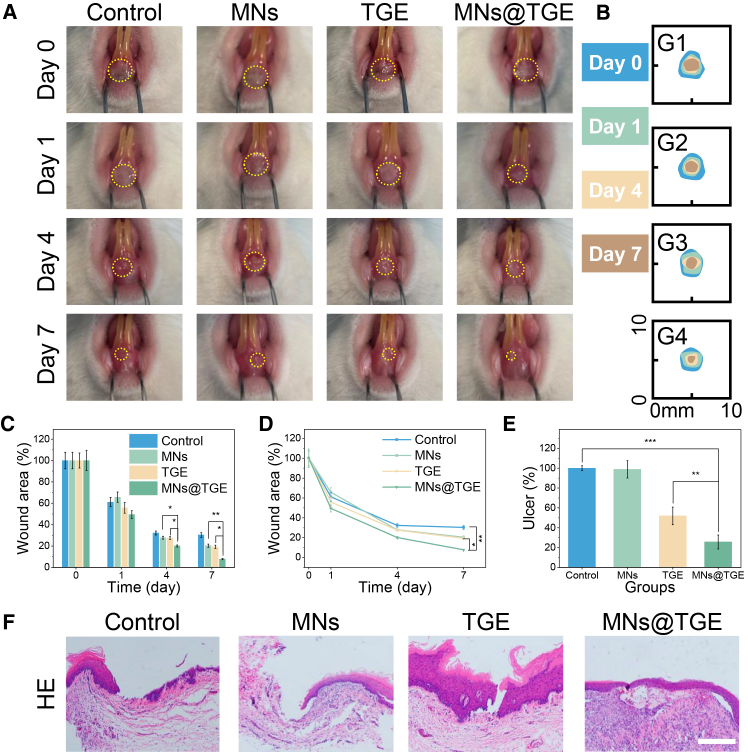
*In vivo* evaluation of the microneedle patch (A) Digital images of the ulcer site wound of the rat oral cavity. (B) Graphic quantization of the wound during the treatment. (C) Bar chart quantifying the ulcer area. (D) Curve graph of the quantified ulcer area. (E) Ulcer area in the different groups at the end of the treatment. *n* = 6; ∗*p* < 0.05; ∗∗*p* < 0.01; ∗∗∗*p* < 0.001. (F) H&E staining images of the wound after therapy. Data are represented as the mean ± SD. Scale bars, 100 μm.

Based on the *in vitro* experiment results, the oral ulcer model was constructed in the rat oral cavity to evaluate the *in vivo* effect. After 7 days, the TGE-integrated microneedle patch-treated group showed the most healing area (Figures 6A and 6B). Compared with the control group, the treated group exhibited the best healing effect during the whole therapeutic period (Figures 6C and 6D). At the end of the treatment, the ulcer area in the TGE-integrated microneedle patch group was found to be significantly smaller than that in the control group and simple drug group (Figure 6E). H&E staining also illustrated that there were obvious epithelial layer defects in the control group. In contrast, the treatment group already had complete epithelial layers (Figure 6F). All these data indicated that the Chinese herbal-integrated microneedle patch could accelerate wound healing at the oral ulcer site.

### Histological analysis of inflammation suppression and tissue regeneration

**Figure 7:**
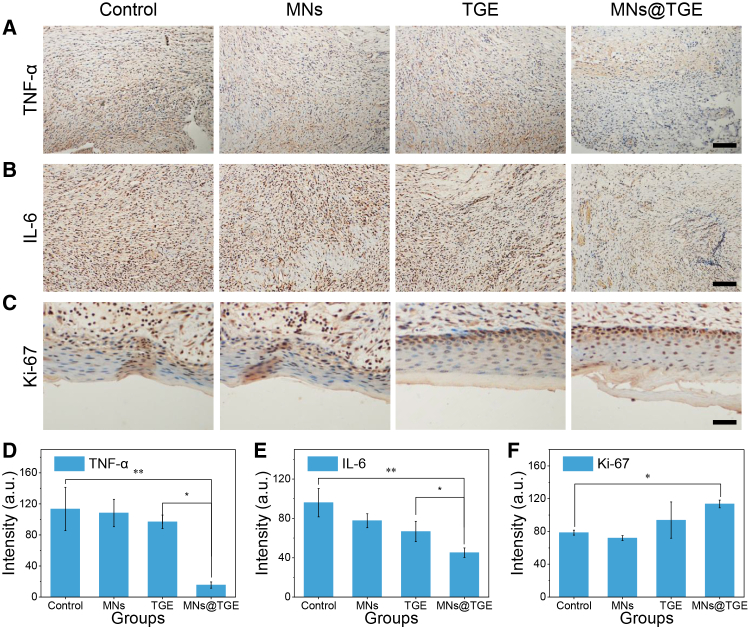
Immunohistochemical staining of the tissue (A) TNF-α staining of the tissue. (B) IL-6 staining of the tissue. (C) Ki-67 staining of the wound site. (D) The quantitated data of TNF-α staining in (A). (E) The quantitated data of IL-6 staining in (B). (F) The quantitated data of Ki-67 staining in (C). Scale bars, 100 μm. Data are represented as the mean ± SD. *n* = 3; ∗*p* < 0.05; ∗∗*p* < 0.01.

The tissue was collected after the experiment to conduct a pathology test for further exploring tissue-level changes in different groups. Tumor necrosis factor (TNF-α) and interleukin 6 (IL-6) were identified as the most representative cytokines in the inflammation of the tissue. The smallest positive area was observed in the treated group through the immunohistochemical (IHC) staining (Figures 7A and 7B). The quantitative data also confirmed the above result (Figures 7D and 7E). The microneedles could deliver the drug more deeply than the powder preparation, thereby alleviating deep-seated inflammation and promoting tissue reconstruction and repair, which is one of the key advantages of microneedles. To evaluate cell proliferation in the epidermal layer, Ki-67 was stained by IHC. Ki-67 is the most popular index showing tissue proliferative level. The Chinese herbal-integrated microneedle patch group tissue showed the highest level in Ki-67 staining, indicating the highest cell proliferation (Figures 7C and 7F). H&E staining of the major organs from the animal did not show a noticeable pathological change in all groups, showing the wonderful biocompatibility of the therapy (Figure S8).

## Discussion

Oral ulcers present as superficial epithelial injuries, yet are frequently accompanied by significant inflammatory responses and elevated ROS levels in the underlying tissues. Conventional topical drug delivery methods are often limited by poor penetration across the mucosal surface. Microneedles, as a representative local drug delivery platform, have shown promising potential to address this limitation. However, their translation into clinical practice for oral applications remains challenging due to the complex oral environment, which includes dynamic factors such as salivary flow that may compromise adhesion and drug retention. What’s more, the initial tissue disruption and discomfort caused by microneedles are a controllable, transient drawback. However, this is counterbalanced by their significant efficacy in targeted drug delivery, which promotes anti-inflammatory responses and tissue regeneration. Consequently, a key direction for future development is to engineer microneedle systems that optimize this therapeutic index through rational design. Traditional Chinese medicine holds significant promise for treating oral diseases, though its associated molecular mechanisms warrant further investigation. With continued advances in materials and design, it is anticipated that an increasing number of conventional drugs and advanced microneedle delivery systems will be developed for targeted oral ulcer therapy.

In summary, we present an octopus sucker-inspired Chinese herbal-integrated microneedle patch that could, *in situ*, release an inner drug to treat oral ulcers. Owing to the sucker-like structure in the patch, the microneedle could be adhered to the wound site of the ulcer. Also, polydopamine enhanced the ability of the patch to rot in the moist oral cavity. Besides, due to the slow release of inner TGE, the microneedle achieved anti-inflammatory and anti-oxidant effects, which alleviated the macrophages’ inflammatory effect and enhanced endothelial cell migration. In addition, the microneedle patch showed an outstanding performance in wound healing and tissue remodeling in the rat oral ulcer model. These properties make the octopus sucker-inspired Chinese herbal-integrated microneedle patch a potential candidate for applications in the treatment of mucosal ulcer and other oral diseases.

### Limitations of the study

This study has several limitations. First, the rats’ oral environment differs from those of humans in terms of salivary flow and tissue mechanics, potentially affecting translational relevance. Second, the manual fabrication process limits scalability and batch consistency. Third, TGE’s complex composition hinders precise quality control and mechanistic elucidation. Fourth, the seven-day observation period captured only early wound healing, leaving long-term safety unexplored. Finally, direct comparison with clinical glucocorticoid formulations was not performed, limiting therapeutic benchmarking. Future large animal studies and standardized manufacturing protocols are needed before clinical translation.

## Resource availability

### Lead contact

Requests for further information and resources should be directed to and will be fulfilled by the lead contact, Chuanhui Song (songchuanhui@smail.nju.edu.cn).

### Materials availability

This study did not generate new unique reagents. All materials generated in this study are listed in the key resources table.

### Data and code availability

• All data reported in this paper will be shared by the lead contact upon request.
• This paper does not report original code.
• Any additional information required to reanalyze the data reported in this paper is available from the lead contact upon request.

## Acknowledgments

We would like to express our gratitude to all those who contributed to this study. We would also like to thank the reviewers and editors for their invaluable input, which has enhanced the quality of this manuscript. This work was supported by the 10.13039/501100001809National Natural Science Foundation of China (82473178 and 82502564), the Jiangsu Provincial Key Research and Development Project (BE2022671), the Medical Research Project of the Jiangsu Provincial Health Commission (ZD2021029), High-Level Hospital Construction Project of Nanjing Stomatological Hospital, Affiliated Hospital of Medical School, Institute of Stomatology, 10.13039/501100008048Nanjing University (0224C002), Jiangsu Provincial Medical Key Discipline (Laboratory), the 10.13039/501100004608Natural Science Foundation of Jiangsu Province (BK20230137 and BK20230142), 10.13039/501100019349Nanjing Medical Science and Technique Development Foundation (YKK23182), and the Startup Grant for Research Assistant, Young Elite Scientists Sponsorship Program by Jiangsu Province Association for Science and Technology (JSTJ-2025-716).

## Author contributions

Conceptualization, C.S., W.H., C.Z., and X.D.; methodology, investigation, and writing – original draft, L.L. and S.G.; writing – review & editing, C.S. and S.G.; funding acquisition, C.S., W.H., C.Z., and L.L.; resources, C.Z. and X.D.; supervision, C.S. and W.H.

## Declaration of interests

The authors declare no competing interests.

## Declaration of generative AI and AI-assisted technologies in the writing process

No generative AI or AI-assisted tools were used for data analysis, content generation, or other aspects, except for grammar and spelling correction.

## STAR★Methods

### Key resources table

**Table:** 

REAGENT or RESOURCE	SOURCE	IDENTIFIER
**Antibodies**		

CD206	Invitrogen	Cat# MA5-16871; AB_2538349
Ki-67	Proteintech	Cat# 28074-1-AP; AB_2918145
iNOS	Proteintech	Cat# 18985-1-AP; AB_2782960
TNF-α	Proteintech	Cat# 17590-1-AP; AB_2271853
IL-6	Proteintech	Cat# 26404-1-AP; AB_3085866
Fluorescein (FITC)–conjugated Goat Anti-Rabbit IgG(H + L)	Proteintech	Cat# SA00003-2; AB_2890897
Rhodamine (TRITC)–conjugated Goat Anti-Rat IgG(H + L)	Proteintech	Cat# SA00007-7;AB_2890953

**Experimental models: Cell lines**		

NIH-3T3	National Biomedical Experimental Cell Repository	STR-authenticated; Mycoplasma-free
RAW264.7	National Biomedical Experimental Cell Repository	STR-authenticated; Mycoplasma-free
HUVECs	National Biomedical Experimental Cell Repository	STR-authenticated; Mycoplasma-free
DPSCs	This paper	N/A

**Experimental models: Organisms/strains**		

Sprague-Dawley Rats	Huachuang Sino	RGD_70508

**Software and algorithms**		

ImageJ	Fiji	N/A
GraphPad Prism	8.4.3	N/A
Origin	2023	N/A
Maxon Cinema 4D	R21	N/A

**Other**		

DCFH-DA ROS probe	Beyotime	Cat# S0033S
Calcein AM	Beyotime	C2012
Hyaluronic acid	Bloomage Biotech	HA-TLM 20-40
LAP (lithium phenyl-2,4,6-trimethylbenzoylphosphinate)	Adamas	CAS: 85073-19-4
Turkish gall	This paper	N/A

### Experimental model and study participant details

#### Animals

Female 8-week-old SD rats (specific pathogen-free grade) were obtained from Huachuang Sino. The animal experiments were approved by the Animal Ethical and Welfare Committee of Nanjing University (IACUC-D2402059). They followed the ‘Guide for the Care and Use of Laboratory Animals’ published by the Chinese National Institutes of Health. Rats were housed under specific pathogen-free (SPF) conditions at 22 ± 2 °C, 50–60% humidity, and a 12-h light/dark cycle, with *ad libitum* access to food and water. All efforts were made to minimize animal suffering. Sex was not assessed as a biological variable, as only female rats were used to reduce variability, which represents a limitation of this study.

#### Rat oral ulcer model

To induce the rat ulcer model, 60% acetic acid was applied to the rat’s mucosa for 3 min under anesthesia. The oral ulcer wound was visible on the second day. The rats were divided into four random groups: 1) control, 2) microneedle, 3) TGE (powder), and 4) microneedle with TGE (6 rats per group). The wound was checked every two days for seven days until the end of the experiment. After treatment, the tissue in the ulcer site and major organs was collected for fixation and paraffin embedding.

### Method details

#### Preparation of Turkish gall extract (TGE)

Turkish gall was crushed and screened by a 50-mesh sieve. Then, 20 g of the screened powder was decocted with 100 mL of 70% (v/v) methyl alcohol three times under ultrasound for 2 h each time, and then placed overnight in the refrigerator at 4 °C. The supernatant was centrifuged at 8000 rpm for 15 min, and then vacuum-concentrated to 50 mL. After freeze-drying, the powder was stored at 4 °C. Before use, it was disinfected by exposing it to ultraviolet light for 30 min.

#### The HPLC analysis of the TGE

The chromatographic analysis was performed using a mobile phase consisting of methanol and 0.5% phosphoric acid, at a flow rate of 0.8–1 mL/min. The injection volume was set at 10–20 μL, and detection was carried out with an ultraviolet detector at a wavelength of 273 nm.

#### Synthesis of the microneedle patch

The Maxon Cinema 4D designed the digital model of the microneedle patch. The data was then input to the 3D printer to get the microneedle patch. The polydimethylsiloxane was used to get the native model of the microneedle patch. The 5% HAMA was mixed with 0.25% LAP; after adding the TGE extracts, the solution was added to the model. By concentrating on removing the bubbles, the light (405 nm) was irradiated for 10s to solidify and get the TGE-loaded microneedles.

#### Characteristics of the microneedle patch

After lyophilization, the MNs were observed by scanning electron microscopy. To observe the structure of MNs, the alkene coupling fluorescent dyes were added to the gels. After solidifying, the confocal laser scanning microscope was used to examine the 3D appearance of the microneedle patch.

#### Fluorescence imaging of drug distribution in mucosa

Under anesthesia, buccal mucosal tissues from rats (both ulcerated and healthy sites) were harvested. Sodium fluorescein powder or sodium fluorescein-loaded microneedles were then applied to the respective mucosal surfaces. Following a brief PBS rinse, the tissue samples were embedded in OCT compound, sectioned, and stained with DAPI for fluorescence microscopy observation. Fluorescence intensity in the lamina propria was quantified using ImageJ.

#### *In vitro* biocompatibility

The dental pulp stem cells (DPSCs), endothelial cells (HUVECs), and Raw264.7 were seeded into the 6-well plates. And the TGE, microneedles, and TGE-loaded microneedles were added to the cell plates. The cells were stained with the living/dead cell fluorescent kit at the selected time point.

#### *In vitro* antioxidant

The hydrogen peroxide (100 μm) simulated the high ROS level. By adding different concentrations of the TGE, the DCFH-DA was used to detect the cellular ROS level of the RAW 264.7. Also, the hydrogen peroxide-treated HUVECs were used to assess the migration ability.

#### *In vitro* anti-inflammation

The LPS-induced Raw264.7 cells showed M1-like macrophages. After being treated with the TGE, the CD206 and iNOS were detected by the IF. Also, the mRNA levels of the relative inflammatory cytokines were detected with qPCR. Sequences of primers are shown in Table S1.

#### IHC staining

The tissue sample embedded in the paraffin was cut into 3 μm slices. After antigen repair, the corresponding antibody was incubated overnight at 4°C. After the secondary antibody was incubated, DAB coloration was used for observation. ImageJ was also used to quantify the images.

### Quantification and statistical analysis

GraphPad Prism software was used to calculate the *p*-value using the Student’s *t* test to compare differences between two groups or one-way analysis of variance (ANOVA), followed by Turkey’s multiple comparisons and two-way ANOVA, followed by Turkey’s multiple comparisons to compare differences between more than two groups. In all cases, statistical differences were considered at ∗*p* < 0.05, ∗∗*p* < 0.01, ∗∗∗*p* < 0.001, ∗∗∗∗*p* < 0.0001, not significant (n.s.), and *p* > 0.05.

## References

[1] Wang Z., Han X., Xiao W., Wang P., Wang J., Zou D., Luo X., Shi L., Wu J., Guo L. (2024). Mussel-inspired adhesive drug-loaded hydrogels for oral ulcers treatment. Acta Biomater..

[2] Xiang Y., Pan Z., Qi X., Ge X., Xiang J., Xu H., Cai E., Lan Y., Chen X., Li Y. (2024). A cuttlefish ink nanoparticle-reinforced biopolymer hydrogel with robust adhesive and immunomodulatory features for treating oral ulcers in diabetes. Bioact. Mater..

[3] Zeng Y., Gao Y., He L., Ge W., Liu J., Yu Y., Xie X. (2023). Multifunctional polysaccharide composited microneedle for oral ulcers healing. Mater. Today Bio.

[4] Sun J., Chen T., Zhao B., Fan W., Shen Y., Wei H., Zhang M., Zheng W., Peng J., Wang J. (2023). Acceleration of Oral Wound Healing under Diabetes Mellitus Conditions Using Bioadhesive Hydrogel. ACS Appl. Mater. Interfaces.

[5] Song C., Lu M., Li N., Gu H., Li M., Lu L., Wang Y. (2025). MXene-Integrated Responsive Hydrogel Microneedles for Oral Ulcers Healing. Smart Med..

[6] Zhu J., Li Y., Xie W., Yang L., Li R., Wang Y., Wan Q., Pei X., Chen J., Wang J. (2022). Low-Swelling Adhesive Hydrogel with Rapid Hemostasis and Potent Anti-Inflammatory Capability for Full-Thickness Oral Mucosal Defect Repair. ACS Appl. Mater. Interfaces.

[7] Zhang Z., Zhang Q., Gao S., Xu H., Guo J., Yan F. (2023). Antibacterial, anti-inflammatory and wet-adhesive poly(ionic liquid)-based oral patch for the treatment of oral ulcers with bacterial infection. Acta Biomater..

[8] Tang Q., Song C., Wu X., Chen H., Yu C., Zhao Y., Qian X. (2024). Dual-functional core–shell microneedle patches for oral ulcers treatment. Chem. Eng. J..

[9] Cui C., Mei L., Wang D., Jia P., Zhou Q., Liu W. (2023). A self-stabilized and water-responsive deliverable coenzyme-based polymer binary elastomer adhesive patch for treating oral ulcer. Nat. Commun..

[10] Song C., Liu R., Fang Y., Gu H., Wang Y. (2024). Developing functional hydrogels for treatment of oral diseases. Smart Med..

[11] Jia B., Zhang B., Li J., Qin J., Huang Y., Huang M., Ming Y., Jiang J., Chen R., Xiao Y., Du J. (2024). Emerging polymeric materials for treatment of oral diseases: design strategy towards a unique oral environment. Chem. Soc. Rev..

[12] Xu Y., Guan J., Wang Q., Xue R., He Z., Lu X., Fan J., Yu H., Turghun C., Yu W. (2023). Mussel-Inspired Caries Management Strategy: Constructing a Tribioactive Tooth Surface with Remineralization, Antibiofilm, and Anti-inflammation Activity. ACS Appl. Mater. Interfaces.

[13] Xue R., He L., Wu J., Kong X., Wang Q., Chi Y., Liu J., Wang Z., Zeng K., Chen W. (2024). Multifunctional sprayable carboxymethyl chitosan/polyphenol hydrogel for wound healing. Int. J. Biol. Macromol..

[14] Luo X., Zha S., Su X., Wang Y., Zhang M., Chen X. (2024). Molecular Basis of the Curative Effects of Turkish Gall Extracts on Periodontitis. ChemistrySelect.

[15] Qi Y., Yang J., Chi Y., Wen P., Wang Z., Yu S., Xue R., Fan J., Li H., Chen W. (2022). Natural polyphenol self-assembled pH-responsive nanoparticles loaded into reversible hydrogel to inhibit oral bacterial activity. Mol. Biomed..

[16] Tu Y., Chen Y., Zheng C., Chen H. (2016). Platelet aggregation promoted by biofilms of oral bacteria and the effect of mouth rinses in vitro. J. Infect. Dev. Ctries..

[17] Zhou X., Zhu D., Wu D., Li G., Liang H., Zhang W., Wu Y., Xu H., Zhang Z., Tong B. (2025). Microneedle delivery of CAR-M-like engineered macrophages alleviates intervertebral disc degeneration through enhanced efferocytosis capacity. Cell Rep. Med..

[18] Cao J., Zhang X., Guo J., Wu J., Lin L., Lin X., Mu J., Huang T., Zhu M., Ma L. (2025). An engineering-reinforced extracellular vesicle–integrated hydrogel with an ROS-responsive release pattern mitigates spinal cord injury. Sci. Adv..

[19] Song C., Zhang X., Lu M., Zhao Y. (2023). Bee Sting-Inspired Inflammation-Responsive Microneedles for Periodontal Disease Treatment. Research.

[20] Song C., Wu X., Wang J., Liu R., Zhao Y. (2023). Photosensitizer-immunotherapy integrated microneedles for preventing tumor recurrence and metastasis. Nano Today.

[21] Luan Q., Qiao R., Wu X., Shan J., Song C., Zhao X., Zhao Y. (2024). Plant-Derived Chinese Herbal Hydrogel Microneedle Patches for Wound Healing. Small.

[22] Shan J., Wu X., Che J., Gan J., Zhao Y. (2024). Reactive Microneedle Patches with Antibacterial and Dead Bacteria-Trapping Abilities for Skin Infection Treatment. Adv. Sci..

[23] Wang G., Wang W., Zhang Z., Wang X., Li S., Choy K.L., Chen Y., Wang Z. (2025). Engineered heterojunction microneedles initiate ROS-mediated “two-hit” mechanism for accelerating impaired wound healing in diabetes. Cell Biomaterials.

[24] Zhang B., Qian Y., Li J., Fan Y., Gao M., Wu Q., Li Z., Hu Y., Du J. (2025). Double-Layer Nucleoside-Microneedles Co-Delivering Dexamethasone Vesicles and Adenosine Overcome Pseudomembrane Barriers for the Treatment of Oral Ulcer. ACS Nano.

[25] Dabbagh S.R., Sarabi M.R., Rahbarghazi R., Sokullu E., Yetisen A.K., Tasoglu S. (2021). 3D-printed microneedles in biomedical applications. iScience.

[26] Ruan S., Li J., Ruan H., Xia Q., Hou X., Wang Z., Guo T., Zhu C., Feng N., Zhang Y. (2024). Microneedle-mediated nose-to-brain drug delivery for improved Alzheimer's disease treatment. J. Control. Release.

[27] Zhu Z., Wang J., Pei X., Chen J., Wei X., Liu Y., Xia P., Wan Q., Gu Z., He Y. (2023). Blue-ringed octopus-inspired microneedle patch for robust tissue surface adhesion and active injection drug delivery. Sci. Adv..

[28] Zhang X., Chen G., Yu Y., Sun L., Zhao Y. (2020). Bioinspired Adhesive and Antibacterial Microneedles for Versatile Transdermal Drug Delivery. Research.

[29] Frey S.T., Haque A.B.M.T., Tutika R., Krotz E.V., Lee C., Haverkamp C.B., Markvicka E.J., Bartlett M.D. (2022). Octopus-inspired adhesive skins for intelligent and rapidly switchable underwater adhesion. Sci. Adv..

[30] Pan G., Li F., He S., Li W., Wu Q., He J., Ruan R., Xiao Z., Zhang J., Yang H. (2022). Mussel- and Barnacle Cement Proteins-Inspired Dual-Bionic Bioadhesive with Repeatable Wet-Tissue Adhesion, Multimodal Self-Healing, and Antibacterial Capability for Nonpressing Hemostasis and Promoted Wound Healing. Adv. Funct. Mater..

[31] Mohammadi Z., Rahsepar M. (2018). Characterization of Mazuj galls of Quercus infectoria tree as green corrosion and scale inhibitor for effective treatment of cooling water systems. Res. Chem. Intermed..

[32] Chen Y., Lei K., Li Y., Mu Z., Chu T., Hu J., Zeng B., Wang Y., Shen J., Cai X. (2025). Synergistic effects of NO/H(2)S gases on antibacterial, anti-inflammatory, and analgesic properties in oral ulcers using a gas-releasing nanoplatform. Acta Biomater..

[33] Gong Y., Wang P., Cao R., Wu J., Ji H., Wang M., Hu C., Huang P., Wang X. (2023). Exudate Absorbing and Antimicrobial Hydrogel Integrated with Multifunctional Curcumin-Loaded Magnesium Polyphenol Network for Facilitating Burn Wound Healing. ACS Nano.

