# Supplementary material for: Octopus sucker-inspired, Turkish gall extract-integrated microneedle patch for oral ulcer mitigation
Source: iScience. 2026 Apr 1;29(5):115557. doi: 10.1016/j.isci.2026.115557 (PMC13092480; doi:10.1016/j.isci.2026.115557)
Supplement: Document S1. Figures S1–S8 and Table S1 [file mmc1.pdf]

iScience, Volume *29*

Supplemental information

Octopus sucker-inspired, Turkish gall extract-integrated microneedle patch for oral ulcer mitigation

Lin Lin, Shilin Guo, Cheng Zhao, Xiaotan Dou, Wei Han, and Chuanhui Song

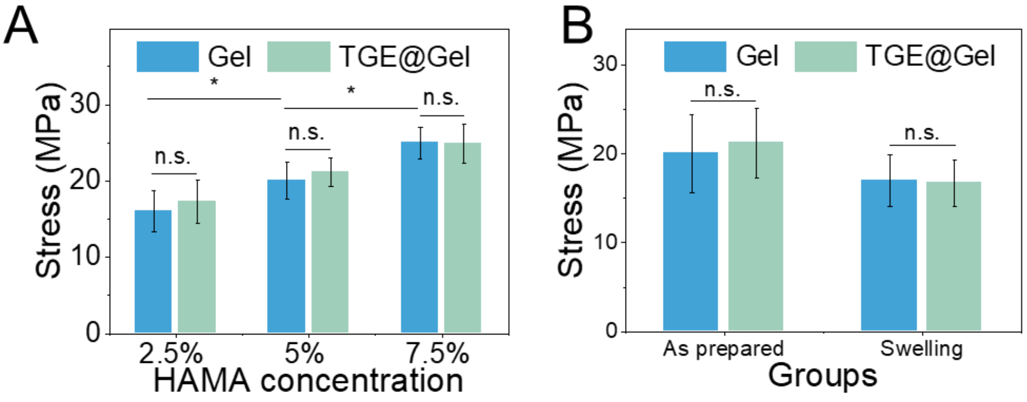

Figure S1. Mechanical characterization of hydrogel formulations.

(A) The mechanical force analysis of different hydrogels.

(B) The mechanical force analysis of hydrogel with/without swelling.

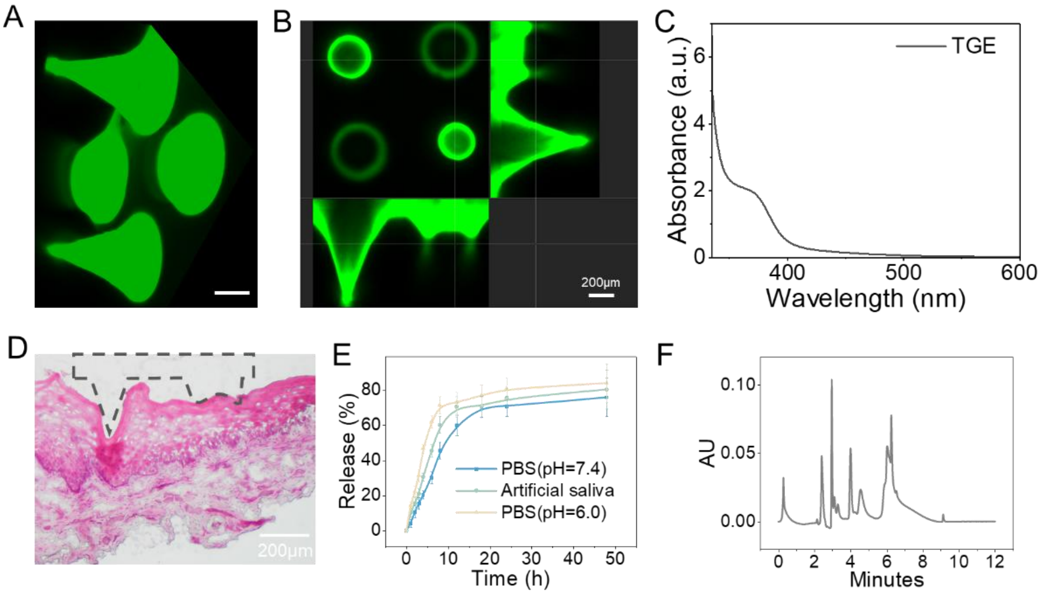

Figure S2. Structural and chemical characterization of the microneedle patch.

(A) Three-dimensional reconstruction of fluorescent dye-labeled microneedle patch showing uniform distribution of tips and suckers.

(B) Cross-sectional view of the microneedle patch confirming structural integrity.

(C) UV-vis absorption spectroscopy analysis of Turkish gall extract (TGE).

(D) HE staining of rat oral mucosa after microneedle insertion, showing penetration depth. Scale bar = 200 μm.

(E) Release profile of TGE from hydrogels under different pH conditions and in artificial saliva.

(F) Representative HPLC chromatogram of TGE.

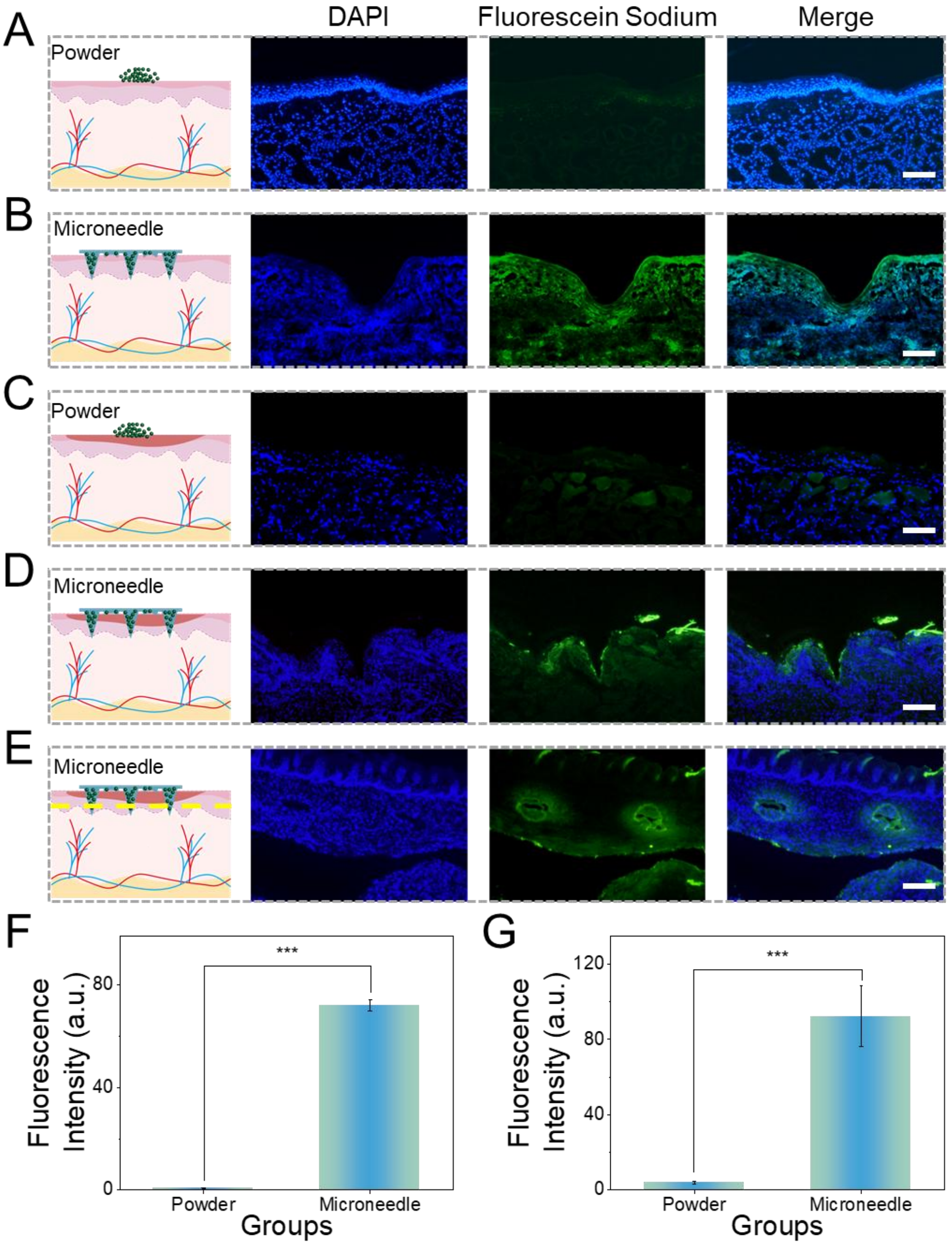

Figure S3. The fluorescence imaging of tissue sections from rat oral mucosa under different treatments.

(A) The fluorescence image of the mucosa with intact epithelial structure after being coated with sodium fluorescein powder.

(B) The fluorescence image of the mucosa with intact epithelial structure after being coated with fluorescein sodium-integrity microneedle.

(C) The fluorescence image of the mucosa with disrupted epithelial structure

after being coated with sodium fluorescein powder.

(D) The fluorescence image of the mucosa with disrupted epithelial structure after being coated with fluorescein sodium-integrity microneedle.

(E) Cross-sectional view of the mucosa with disrupted epithelium treated with fluorescein sodium-loaded microneedle.

(F) The quantitative analysis of the fluorescent intensity of the lamina propria in A) and B).

(G) The quantitative analysis of the fluorescent intensity of the FITC in the lamina propria in C) and D). n=3, ***$p < 0.001$, scale bar=100μm.

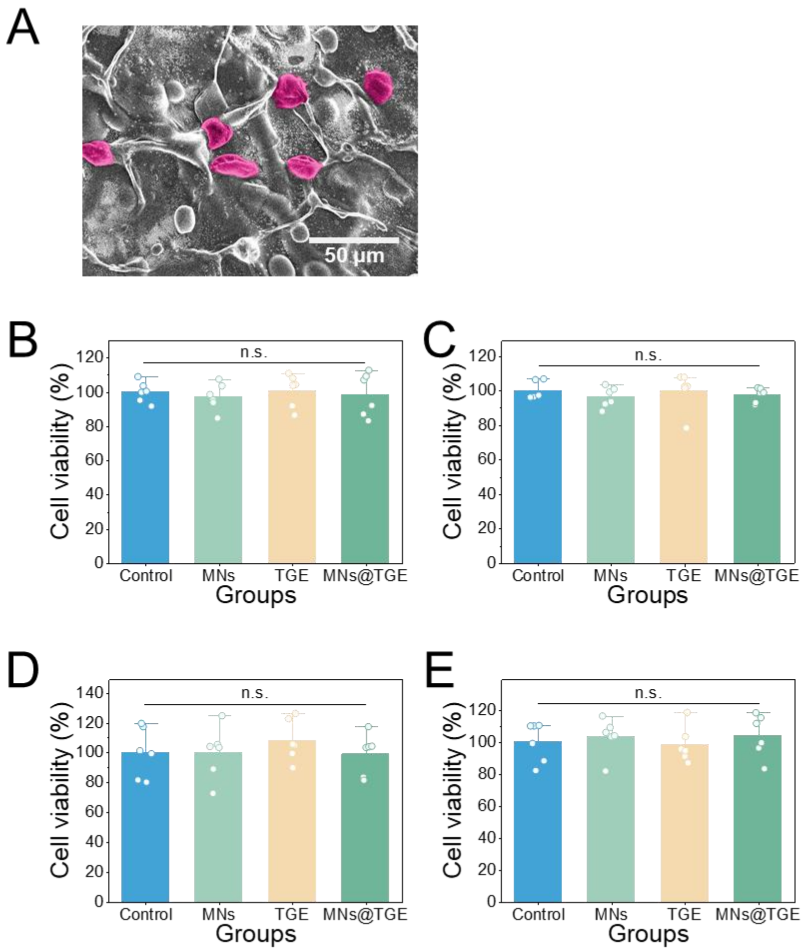

Figure S4. Biocompatibility evaluation of the microneedle patch.

(A) The SEM of the HUVECs' growth status on the hydrogel. Scale bar = 50 μm.

(B) CCK-8 assay of DPSCs cultured with different treatments.

(C) CCK-8 assay of HUVECs under the same conditions.

(D) MTT assay of DPSCs with different treatments.

(E) MTT assay of HUVECs with different treatments. N=6, n.s., not significant.

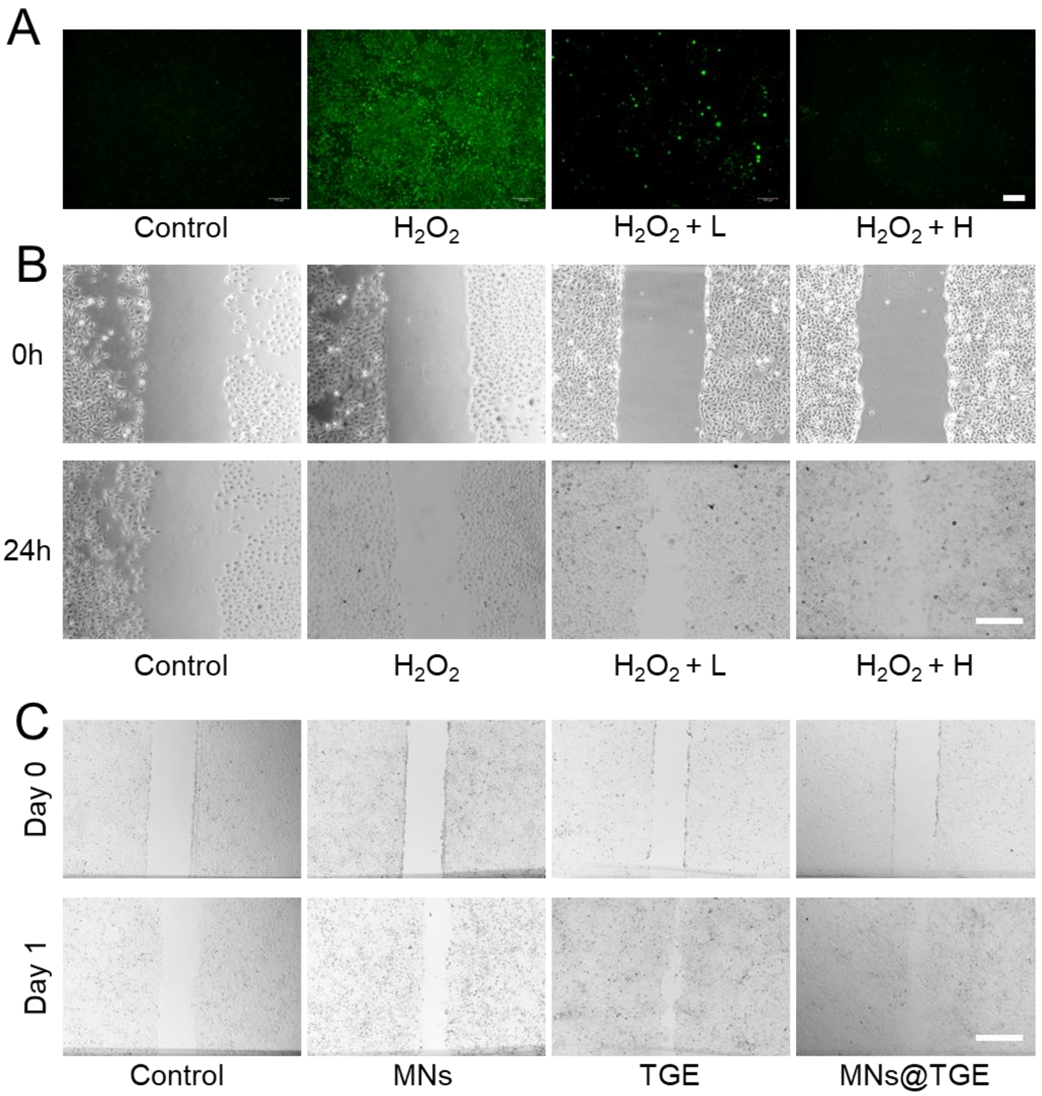

Figure S5. In vitro antioxidant and migration assays.

(A) The DCFH-FA fluorescent staining of the Raw264.7 with high ROS.

(B) The scratch healing experiments of the HUVECs with high ROS level with or without the TGE.

(C) The origin figures of the Figure 5A. Scale bar=100μm.

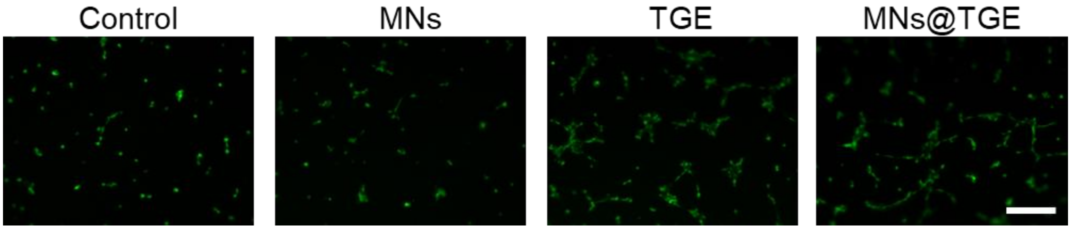

Figure S6. Tube formation assay of HUVECs.

The fluorescent staining of the HUVECs tube formation. Scale bar=100μm.

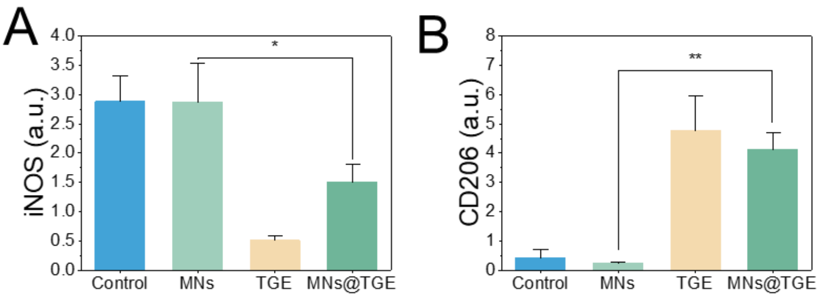

Figure S7. Quantitative analysis of macrophage polarization, Related to Figure 5C.

(A) The quantized data of the iNOS staining of the Raw264.7.

(B) The quantized data of the CD206 staining of the Raw264.7.

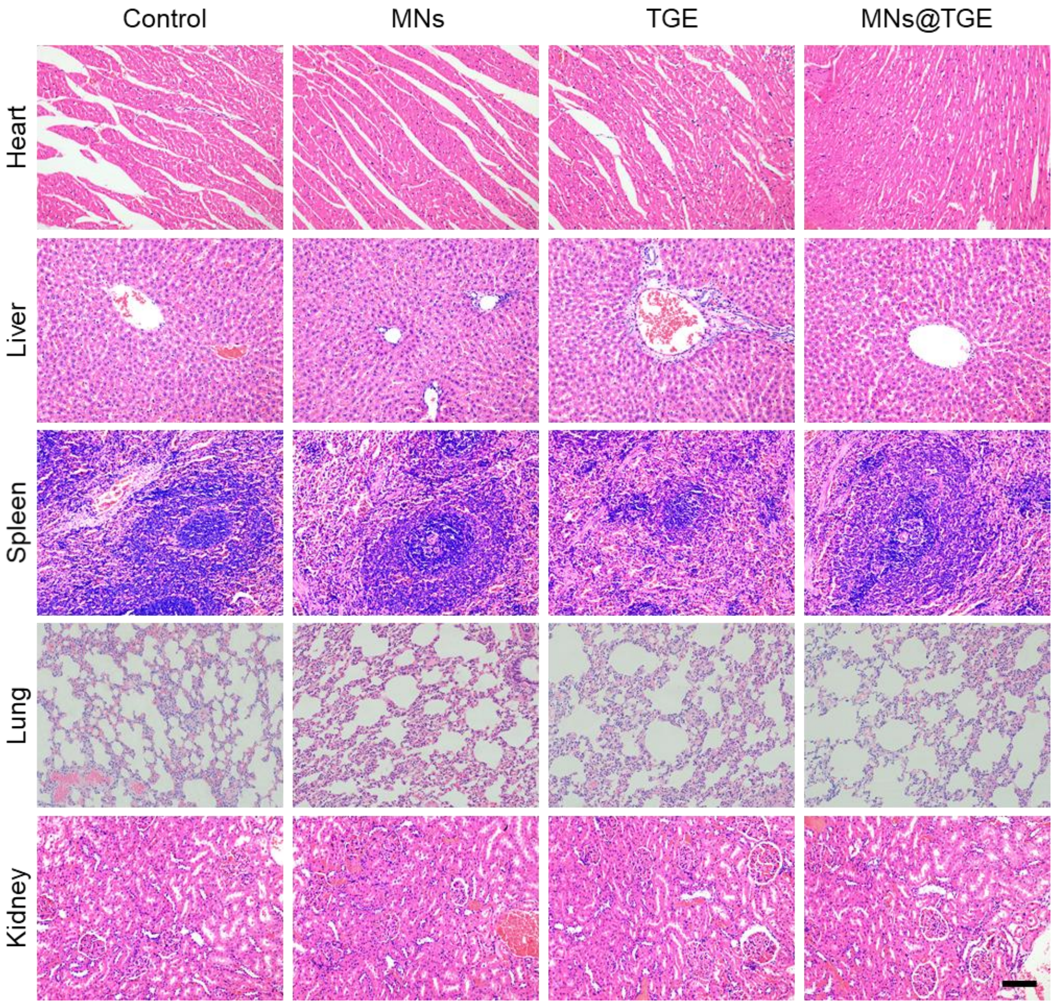

Figure S8. Biosafety evaluation in major organs.

The HE staining of the major organs of the rats. Scale bar=100μm.

Table S1: Primer sequences used in this study

TNF-α-F	AGGCACTCCCCCAAAAGATG
TNF-α-R	CCACTTGGTGGTTTGTGAGTG
iNOS-F	TTCTCAGCCACCTTGGTGAAG
iNOS-R	GCTACTCCGTGGAGTGAACA
IL-1β-F	TGCCACCTTTTGACAGTGATG
IL-1β-R	TGTGCTGCTGCGAGATTTGA
IL-6-F	TAGTCCTTCCTACCCCAATTTCC
IL-6-R	TTGGTCCTTAGCCACTCCTTC
beta-actin-F	CATTGCTGACAGGATGCAGAAGG
beta-actin-R	TGCTGGAAGGTGGACAGTGAGG
